# Unusual dynamic precipitation softening induced by dislocation glide in biomedical beta-titanium alloys

**DOI:** 10.1038/s41598-017-08211-7

**Published:** 2017-08-14

**Authors:** Koji Hagihara, Takayoshi Nakano, Mitsuharu Todai

**Affiliations:** 1 Department of Adaptive Machine Systems, Graduate School of Engineering, Osaka University, Suita, Osaka 565-0871 Japan; 2 Division of Materials and Manufacturing Science, Graduate School of Engineering, Osaka University, Suita, Osaka 565-0871 Japan

## Abstract

Softening of metallic materials containing precipitates during cyclic deformation occurs through dissolution of the precipitates, because the to-and-fro motion of the dislocation causes dissolution of the precipitate particles by cutting them. Here, however, we found the completely opposite phenomenon for the first time; a “dynamic precipitation softening” phenomenon. In a Ti-35Nb-10Ta-5Zr body-centered cubic structured β-Ti alloy single crystal developed for biomedical implant, the to-and-fro motion of the dislocation “induced” the selective precipitation of the ω-phase whose *c*-axis is parallel to the Burgers vector of the moving dislocation, which led to the significant cyclic softening of the crystal. The formation of the ω-phase is generally believed to induce significant hardening of β-Ti alloys. However, the present results suggest that this is not always true, and control of the anisotropic features of the ω-phase via control of crystal orientation can induce unusual mechanical properties in β-Ti alloys. The unique anisotropic mechanical properties obtained by the cyclic-deformation-induced oriented ω-phase formation could be useful for the development of “single-crystalline β-Ti implant materials” with advanced mechanical performance.

## Introduction

The importance of developing biomaterials has been growing as the elderly population increases in our society^1^. To improve the hard tissue replacements used as artificial hip joints, bone plates, dental implants, and so on, β-type titanium alloys with a body-centered cubic (bcc) structure have received much attention^2–4^. Among them, Ti-Nb-Ta-Zr quaternary alloys are especially interesting because of their superior properties such as high specific strengths and low cytotoxicity^5–9^. In addition, their extremely low Young’s moduli of approximately 60–80 GPa in the polycrystalline form make them attractive for hard tissue replacement, because reducing the Young’s modulus of the implant material to a value comparable to that of human bones (20–40 GPa) is extremely desirable in order to overcome the problem of spontaneous bone absorption around the hard tissue replacements due to so-called “stress-shielding”^10, 11^. Moreover, it has been clarified that control of the crystal orientation in some β-Ti alloys enables their Young’s moduli to be decreased to ~30–45 GPa^12–16^, which is comparable to that of human bone^17^. Because of this attractive property, we expect the development of a “single-crystalline β-Ti implant material”^18, 19^ that can significantly reduce stress-shielding and suppress spontaneous bone adsorption, as single-crystalline Ni-based superalloys are used in turbine blades in jet engine to achieve the maximum potential of that material^20, 21^.

For the practical application of this single-crystalline β-Ti alloy as an implant material, its fatigue properties must be understood, because most hard tissue replacements are used under severe cyclic loading conditions. In this research, we first found that very curious cyclic softening behavior occurs in some β-Ti alloy single crystals, accompanied by dynamic precipitation of the ω-phase with a hexagonal unit cell. This is completely opposite to what occurs in materials in general, where dissolution of precipitates causes cyclic softening because the to-and-fro motion of the dislocation cuts the precipitates and assists in their dissolution. In this paper, we discuss the origin of this curious cyclic softening behavior accompanied by the precipitation of the ω-phase.

## Results

### Dynamic precipitation of ω-phase followed by the cyclic softening

**Figure 1:**
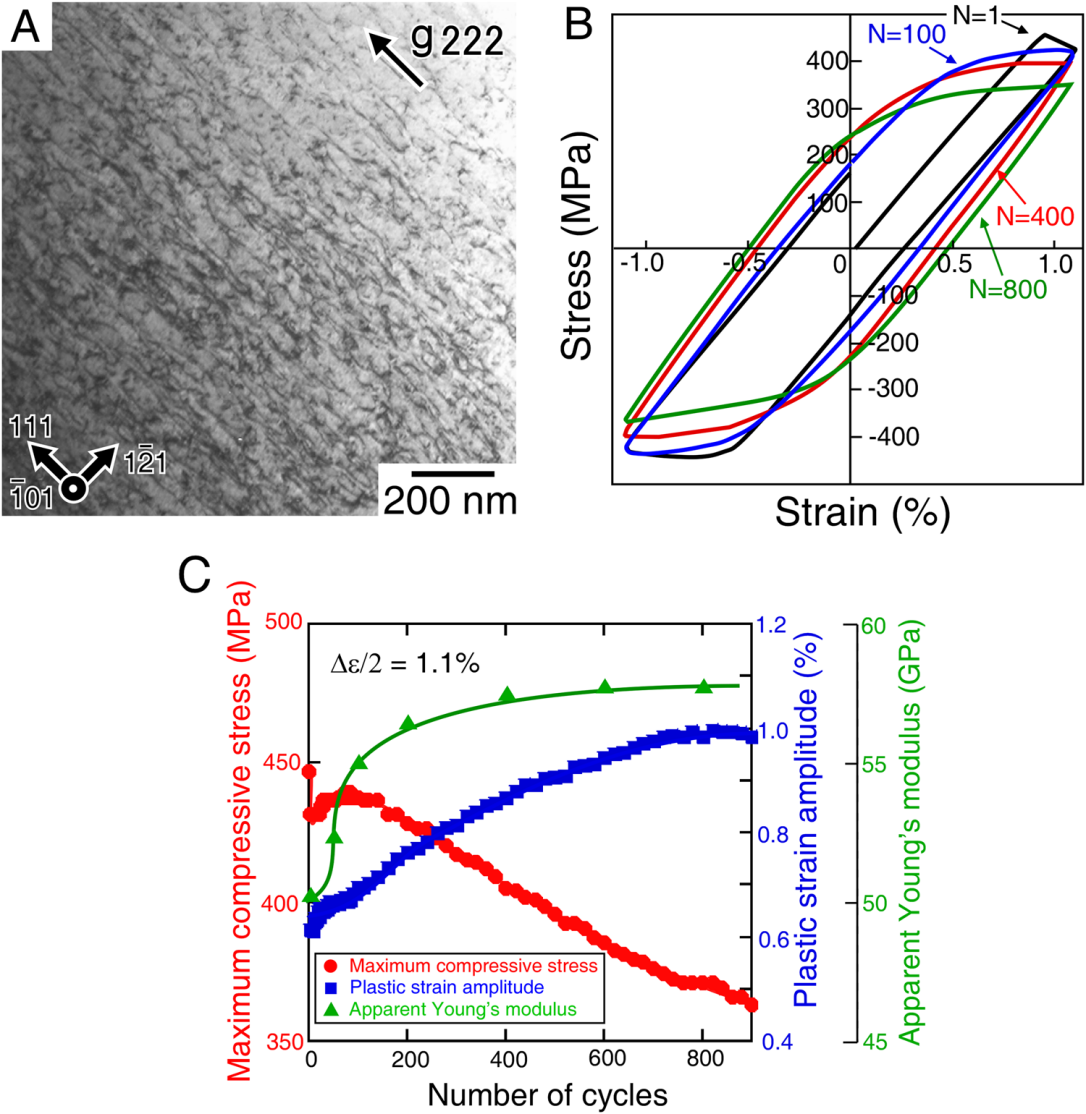
Cyclic deformation behavior of a Ti-35Nb-10Ta-5Zr (wt. %) single crystal in which dislocations are operative. (**A**) TEM bright-field images of the dislocations observed in the specimen cyclically deformed at Δε/2 = 1.1% to ~920 cycles. Beam//[10$\bar{1}$]. Dislocations with the Burgers vector parallel to [111] were operative as screw dislocations. (**B**) Typical hysteresis loops for a controlled total strain amplitude Δε/2 of 1.1% applied at the [$\bar{1}$49] loading orientation. (**C**) Variations in maximum stress, plastic strain amplitude, and apparent Young’s modulus with the number of cycles.

The material examined in this study was a single-crystalline Ti-35Nb-10Ta-5Zr (wt. %) alloy, and tension-compression cyclic deformation tests were conducted. It is known that several kinds of deformation modes such as stress-induced martensitic transformation, {332} <113> twinning, and/or {101} <111> slip are operative in β-Ti alloys, depending on their alloy composition^22, 23^, but in this alloy we confirmed that only {101} <111> slip was operative during the cyclic deformation, as shown in Fig. 1A.

Figure 1B shows typical hysteresis loops of a specimen cyclically deformed at the [$\bar{1}$49] orientation at a controlled total strain amplitude of Δε/2 = 1.1% at ambient temperature. For this loading orientation, dislocations with the Burgers vector parallel to the [111] direction were only operative on the (10$\bar{1}$) slip plane, because it had the largest Schmid factor of 0.500 (Supplementary Table S1). This was indeed confirmed by the optical microscope analysis and transmission electron microscope (TEM) observation (Fig. 1A and Supplementary Fig. S1). The positive and negative stress values in the hysteresis loops shown in Fig. 1B indicate tensile and compressive stress, respectively. The loops show conventional leaf-like shapes, as observed in many metallic materials, but the maximum stress and plastic strain amplitude in the loops exhibit curious variations as the cyclic deformation proceed. Figure 1C shows the variations in maximum stress, plastic strain amplitude, and apparent Young’s modulus of the specimen during cycling. The maximum stress dropped largely in the second cycle because of the appearance of a yield drop in the first cycle owing to the escape of grown-in dislocations from the impurities atmosphere. This is known as the Cottrell effect^24^. After the second cycle, the maximum stress gradually increases and then remains almost constant up to approximately 100 cycles. After ~100 cycles, however, the maximum stress gradually decreases as the number of cycles increases, and the plastic strain amplitude in the hysteresis loops increases as well. In other words, significant cyclic softening appeared. It is plausible that initial cyclic hardening followed by cyclic softening or the appearance of a stress plateau occurs by rearrangement of the dislocations. In the present case, however, the maximum stress continuously decreased as the number of cycles increased after ~100 cycles, and the maximum stresses exhibited the lower values than the yield stress of the undeformed specimen. Since the dislocation density was quite low in the as-grown single crystal, the decrease in maximum stress to values lower than the initial yield stress cannot be explained only by rearrangement of the dislocations. Instead, it should be noted that the apparent Young’s modulus rapidly increased during the initial 200 cycles, which is the region before the cyclic softening began to be significant. The dramatic variations in mechanical properties accompanied by the increase in Young’s modulus imply that changes in the microstructure occurred during the cyclic deformation.

**Figure 2:**
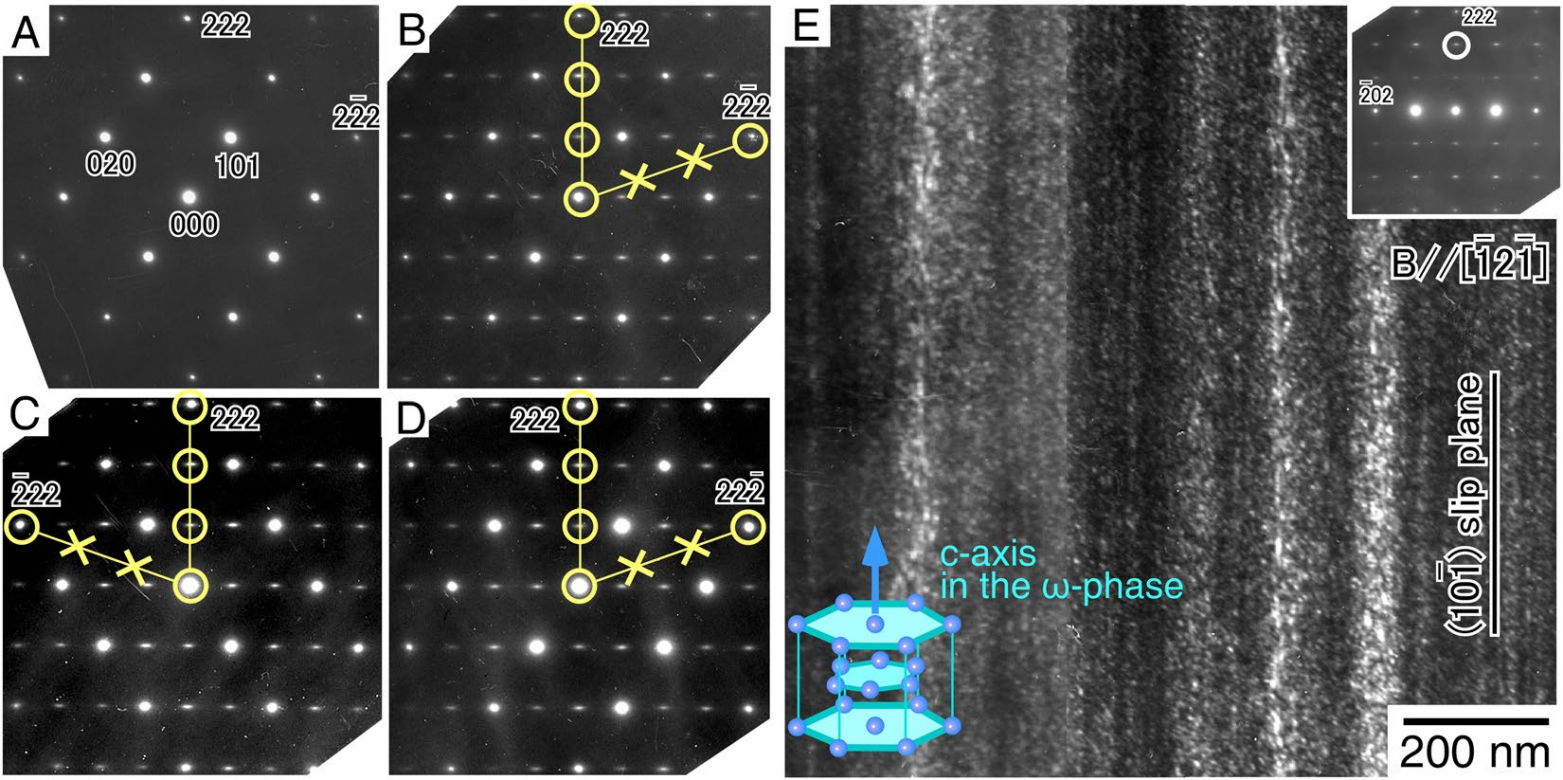
Curious ω-phase formation induced by the cyclic deformation. (**A**) SAED pattern in undeformed specimen observed along the [10$\bar{1}$] direction. (**B–D**), SAED patterns of the specimen cyclically deformed at Δε/2 = 1.1% to ~920 cycles, observed along the [10$\bar{1}$], [01$\bar{1}$], and [$\bar{1}$10] directions, respectively. The extra spots derived from the ω-phase appear along only one of the four <111> directions. (**E**) Dark-field image showing the abundant precipitation of the ω-phase, observed along the [$\bar{1}$2$\bar{1}$] direction. The extra spot used for the observation is denoted by the circle in the SAED pattern.

The occurrence of such changes was actually confirmed by TEM observations. Figure 2A shows the selected area electron diffraction (SAED) pattern of the undeformed specimen observed along the [10$\bar{1}$] direction. Only the fundamental spots derived from the bcc β-matrix phase are observed, and the absence of any precipitate is also confirmed in the bright-field observation. Nevertheless, in the specimen cyclically deformed up to fracture (*N* = ~920 cycles), many extra spots surprisingly appeared along the [111] direction between the spots of the β-matrix phase, as shown in Fig. 2B. The positions of the extra spots were wholly coincident with those derived from the ω-phase with a hexagonal unit cell. Figure 2E shows the dark-field image of the cyclically deformed specimen observed along the [$\bar{1}$2$\bar{1}$] direction, which is perpendicular to the (10$\bar{1}$) slip plane. Abundant precipitates with tiny dot-like contrast are indeed observed. They tended to collectively form on a particular (10$\bar{1}$) plane, parallel to the slip plane of the dislocations.

SAED patterns were also observed along the [01$\bar{1}$] and [$\bar{1}$10] directions, as shown in Fig. 2C,D, respectively. These patterns confirmed that, curiously, the extra spots derived from the ω-phase only appeared along one of the four <111> directions. The [111] direction, along which the extra spots appeared, was parallel to the Burgers vector of the moving dislocation, and no extra spots were seen along the other [$\bar{1}$11], [1$\bar{1}$1], and [11$\bar{1}$] directions. This is inconsistent with general knowledge about the ω-phase. The ω-phase is known to precipitate in β-Ti alloys under annealing at relatively low temperatures or upon quenching from high temperatures. In those cases, the ω-phase precipitates with a distinct crystal orientation relationships relative to the β-matrix phase, as follows^25–27^:

1 $${\{111\}}_{{\rm{\beta }}}//{(0001)}_{{\rm{\omega }}}, < 1\bar{1}0{ > }_{{\rm{\beta }}}// < 11\bar{2}0{ > }_{{\rm{\omega }}}$$

Thus, equivalent amounts of four variants of ω-phases always precipitate, such that the [0001] *c*-axis in the ω-phase is parallel to the [111], [$\bar{1}$11], [1$\bar{1}$1], or [11$\bar{1}$] axes of the β-matrix phase.

In contrast, surprisingly, the present analyses shown in Fig. 2B–D demonstrate that only one variant of the ω-phase forms in a cyclically deformed specimen whose *c*-axis is parallel to the [111] direction, which is parallel to the Burgers vector of the operative dislocation. This suggests that the ω-phases were formed not through the effect of heat, but were dynamically precipitated under cyclic deformation, i.e., because of the to-and-fro motion of the dislocations, accompanied by unusual softening. The increase in the apparent Young’s modulus during the cyclic deformation must correspond to the increase in the amount of ω-phase. Increases in the Young’s modulus owing to the precipitation of ω-phases have been reported in many β-Ti alloys^6, 28, 29^.

We further confirmed that the same behavior, cyclic softening accompanied by the formation of the ω-phase, also occurs in binary Ti-Nb single crystals (Supplementary Figs S2 and S3). Thus, this softening behavior is not restricted to the Ti-35Nb-10Ta-5Zr ternary crystal used in the current study; rather, it must be a more general behavior occurring in many β-Ti single-crystalline alloys in which dislocations are operative.

**Figure 3:**
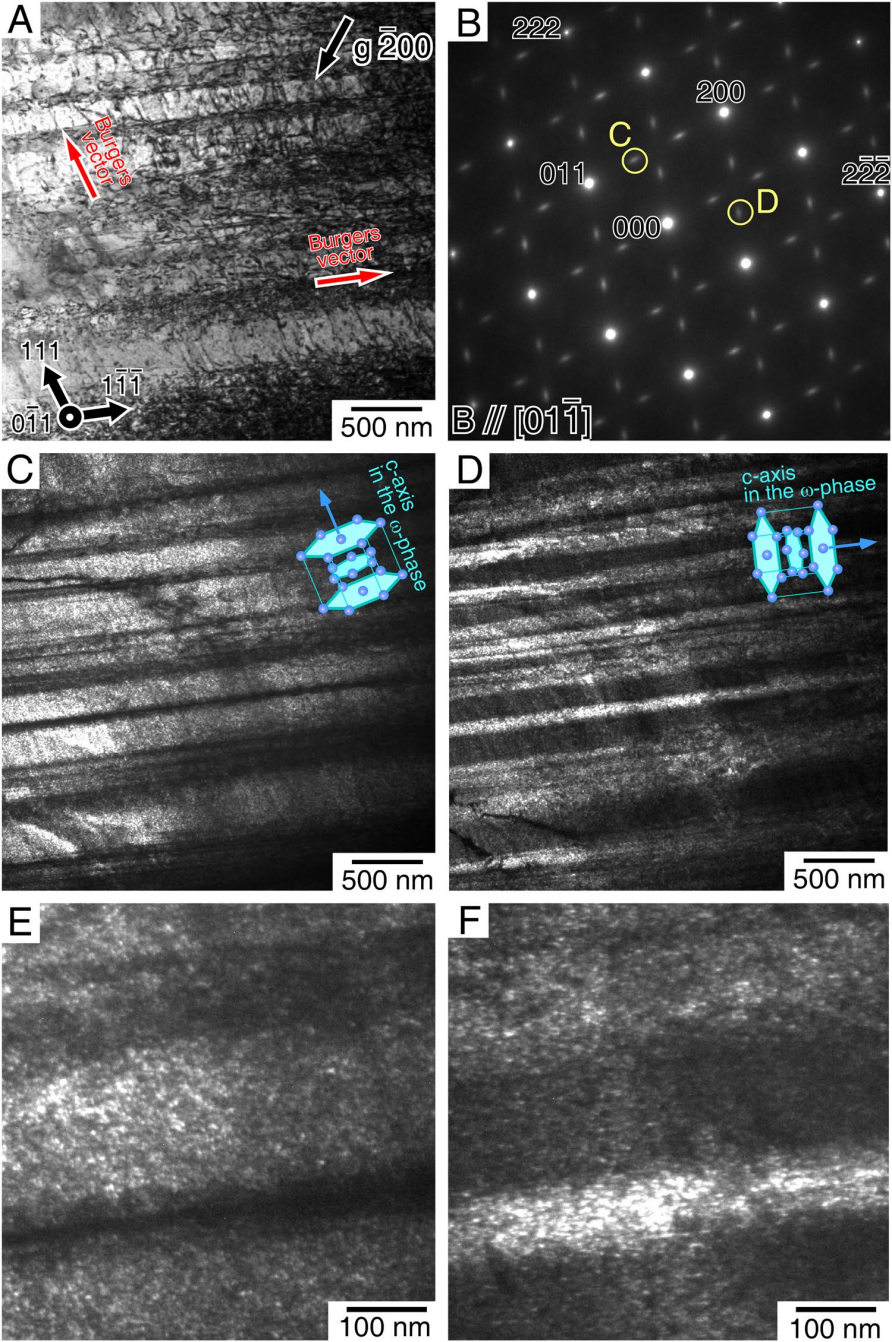
Formation of two ω-phase variants in the specimen in which two kinds of dislocations are operative. (**A**) Dislocation morphology in the specimen cyclically deformed at the [012] orientation at Δε/2 = 1.1% to ~830 cycles. Beam//[01$\bar{1}$]. Two kinds of dislocations with the Burgers vector parallel to [111] and [$\bar{1}$11] were observed to be operative as screw dislocations. (**B**) SAED pattern in the cyclically deformed specimen. The extra spots used for dark-field observations shown in Fig. 3C,D are denoted by the yellow circles. (**C**,**D**) Dark-field images showing the ω-phases observed in the same area shown in Fig. 3A. Two kinds of ω-phases appeared in separate regions, obviously following the dislocation distribution. (**E,F**) Higher magnification images of the precipitated ω-phase particles shown in Fig. 3C and D, respectively.

The formation of ω-phases because of cyclic deformation was further confirmed by a cyclic deformation test at a different loading orientation of [012]. In this loading orientation, the Schmid factors for the (10 $\bar{1}$)[111] and (101)[$\bar{1}$11] slips exhibit the same high values of 0.490 (Supplementary Table S1). Therefore, in the cyclic deformation, the two kinds of dislocations with different Burgers vectors were expected to be equally operative. Figure 3A shows a bright-field TEM image of a specimen cyclically deformed to fracture (*N* = ~830). Two kinds of dislocations aligned parallel to [111] and [$\bar{1}$11] were observed in the specimen. By g·b contrast analysis, the Burgers vectors of the dislocations were confirmed to be [111] and [$\bar{1}$11], as expected (Supplementary Fig. S4). The [111] and [$\bar{1}$11] dislocations had screw characters, and they tended to exist in separate regions. Figure 3B shows the corresponding SAED pattern taken at the region shown in Fig. 3A. The observed SAED pattern is different from that shown in Fig. 2, suggesting that the precipitation behavior of the ω-phases varied in the specimen depending on the loading orientation for the cyclic deformation. This was clearly confirmed by the dark-field images of the same area shown in Fig. 3C and D. In contrast to the former [$\bar{1}$49] specimen in which only [111] dislocations were operative, this specimen exhibited two kinds of ω-phases in separate regions that obviously followed the dislocation distribution. Figure 3E and F show higher-magnification images of the precipitated ω-phase particles. By careful observations, the directions of the *c*-axes of the ω-phase in the precipitates were confirmed to correspond to the direction of the Burgers vector of the dislocations dominantly operative in the same regions. This provides evidence that the dislocation motion dynamically induces the precipitation of the ω-phase.

It is noteworthy that the deformation microstructure was also observed in the specimen cyclically deformed at Δε/2 = 0.7% in which only elastic deformation occurred during the test. In the specimen, however, the dynamic precipitation of ω-phases was not observed even after ~9000 cycles (Supplementary Figs S5A and B). This suggests that the formation of the ω-phase was not induced simply by the applied stress; rather the to-and-fro motion of the dislocation caused by cyclic deformation must be related to the ω-phase formation. This is the first finding of such curious dynamic precipitation behavior induced by dislocation glide in materials, as far as we know. Moreover, this dynamic precipitation led to curious cyclic softening of the alloy, since there was little precipitation in the specimen before softening (at 50 cycles, Supplementary Fig. S5C). However, precipitation was significant in further cyclically deformed specimens in which significant softening appeared, as shown in Figs 2 and 3. Furthermore, no cyclic softening was observed in the specimen cyclically deformed at Δε/2 = 0.7%, in which dynamic precipitation of ω-phases did not occur (Supplementary Fig. S6).

### Unique “unidirectional softening” induced by dynamic precipitation of the ω-phase

**Figure 4:**
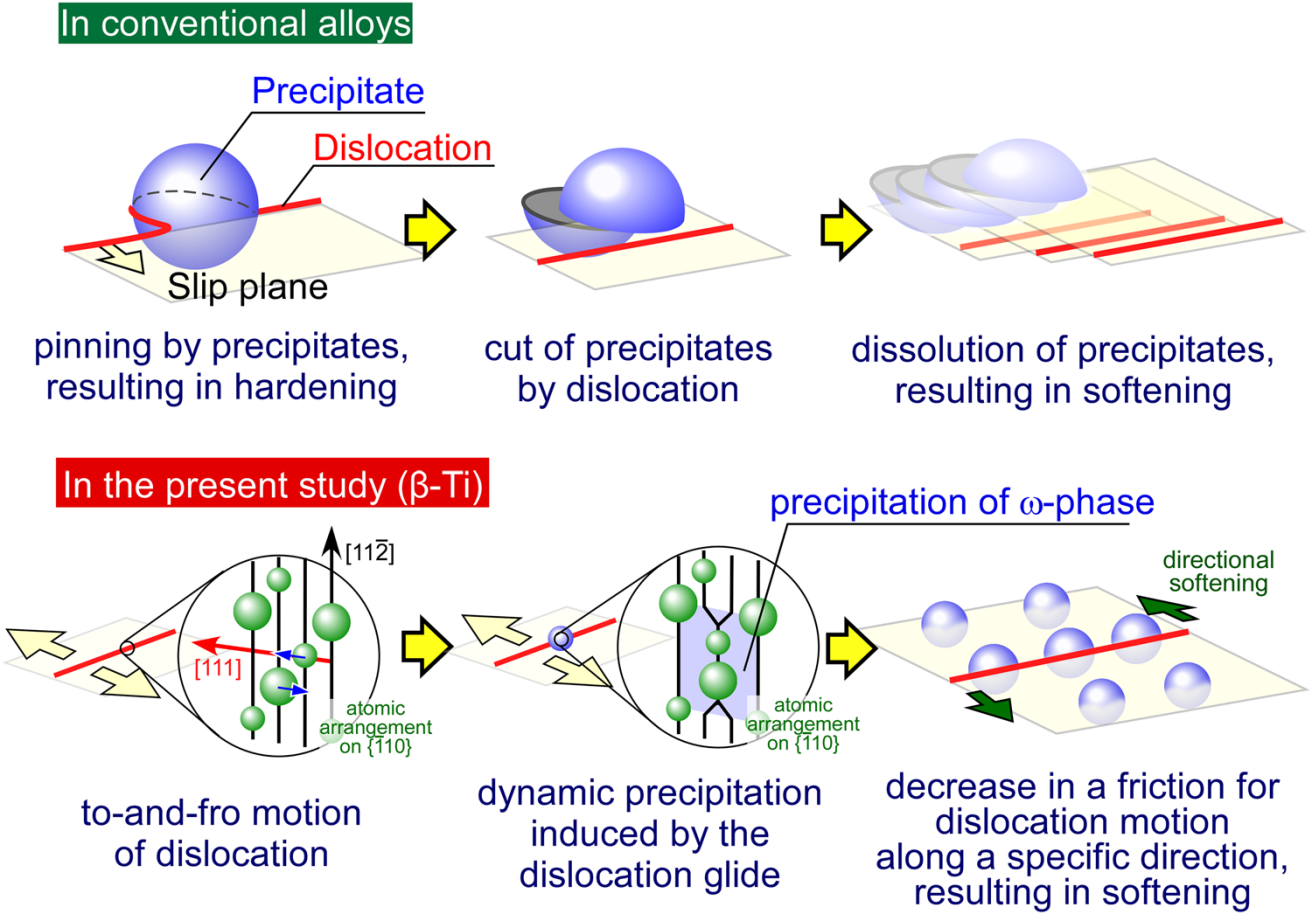
The difference in the softening mechanisms of the alloys induced by cyclic deformation, in conventional alloys and in the β-Ti alloys which was first found in the present study. On the detailed mechanism of the dynamic precipitation of ω-phase by dislocation glide, further explanation is available in Fig. 6.

As described in the introduction, there have been many reports on the cyclic softening of metallic materials that contain precipitates, including Ti alloys^30–34^. In these materials, the softening is known to occur through dissolution of the precipitates because the to-and-fro motion of the dislocation cuts the precipitates and assists in their dissolution, as schematically indicated in Fig. 4. In contrast, the present phenomenon is completely reversed, in that the to-and-fro motion of the dislocations “induces” the precipitation of ω-phases, and cyclic softening follows. This is also counter to generally accepted knowledge about the ω-phase, i.e., that ω-phase precipitation induced by conventional annealing strongly hardens β-Ti alloys and increases their fatigue strength^6, 22, 35^. This unusual behavior is expected to be derived from the strong anisotropic mechanical properties of the ω-phase. This was proved in this study by using the above-mentioned cyclically deformed specimen.

**Figure 5:**
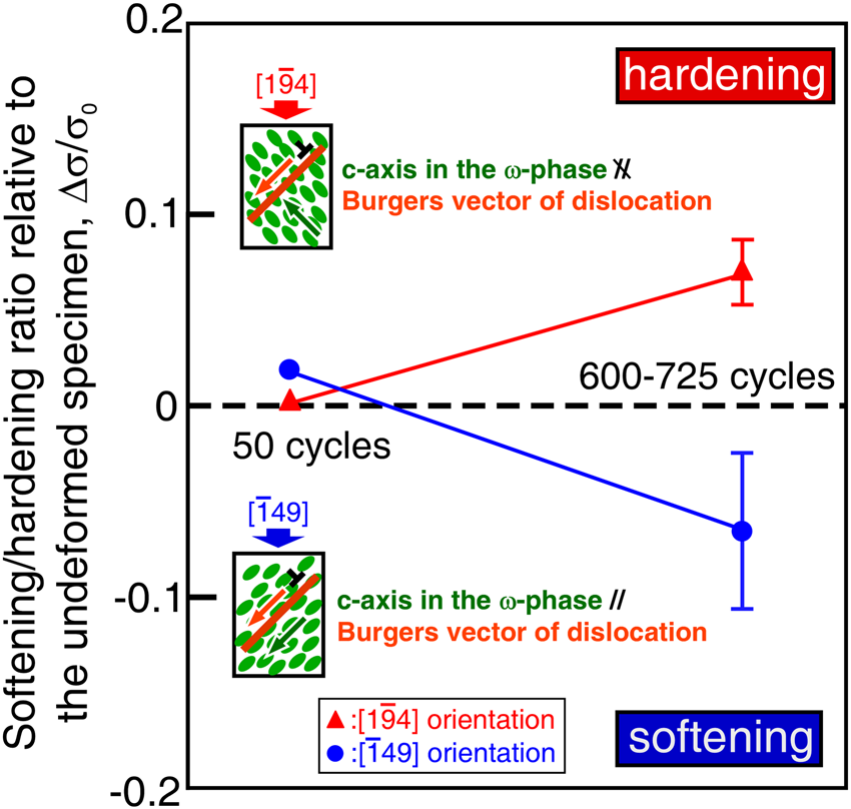
Variation in yield stress owing to the formation of ω-phases depending on the loading orientation. The yield stresses of specimens cut out from specimens cyclically deformed at Δε/2 = 1.1% to 50 cycles and 600–725 cycles, were examined at the [$\bar{1}$49] and [1$\bar{9}$4] loading orientations. The vertical axis value indicates the softening/hardening ratio relative to the undeformed specimen, Δσ/σ_0_, where σ_0_ is the yield stress of the undeformed specimen and Δσ is the difference in yield stress between the previously cyclically deformed specimen and the undeformed specimen. The positive and negative values indicate that hardening and softening are induced by the cyclic deformation, respectively.

For this purpose, small rectangular specimens were cut out from the specimen cyclically deformed at Δε/2 = 1.1% to 600–725 cycles, where strong cyclic softening occurred as shown in Fig. 1C, and the yield stress of the cut sample was examined by using a monotonic compression test. In this compression test, two loading orientations were selected. One was parallel to [$\bar{1}$49], which was parallel to the loading orientation in the previous cyclic deformation, and hence the *c*-axes of the ω-phases were aligned along [111], that is parallel to the Burgers vector of the operative dislocations. For the other specimens, the loading orientation was set to be parallel to [1$\bar{9}$4], in which the *c*-axes of the ω-phases were at an angle of 70.5° to the Burgers vector of the operative [11$\bar{1}$] dislocations. Specimens with the same geometry were also cut out from the undeformed single crystal and from a specimen cyclically deformed for 50 cycles, in which the dislocations were introduced but the one-variant ω-phase was not yet significantly developed (Supplementary Fig. S5C). The [$\bar{1}$49] and [1$\bar{9}$4] specimens had the same Schmid factors of 0.500 for the {10$\bar{1}$} <111> slip, and hence the yield stress exhibits the same value in the undeformed specimens. Figure 5 shows the variation in the yield stresses of the specimens relative to that of the undeformed specimen, which was taken as the hardening/softening ratio. For the specimen cut out from the specimen deformed for 50 cycles, the yield stress was almost the same as that of the undeformed specimen, and the difference between the yield stresses of the specimens with the [$\bar{1}$49] and [1$\bar{9}$4] loading orientations was quite small. This indicates that the influence of the morphology of the dislocation introduced during the cyclic deformation on the yield stress was small. In contrast, for the specimen cut out from the specimen deformed for 600–725 cycles, there was a large difference in yield stress between the [$\bar{1}$49] and [1$\bar{9}$4] loading orientations. In the specimen deformed parallel to [$\bar{1}$49], the yield stress decreased significantly relative to that of the undeformed specimen, reflecting the cyclic softening. On the other hand, in the specimen deformed parallel to [1$\bar{9}$4], the yield stress was slightly higher than that of the undeformed specimen. This demonstrates that although softening of the specimen was induced by cyclic deformation, the softening occurred only in a specific direction in the crystal; in the other directions, the specimen was hardened by the precipitation of the ω-phase. That is, dynamic precipitation of ω-phase accompanied by the specific selection of one of four variants induced curious unidirectional softening of the crystal.

## Discussion

As described above, we found dynamic precipitation of the ω-phase and the following curious unidirectional softening in β-Ti alloys. As far as we know, this is the first report on such a “dynamic precipitation softening” in metals.

**Figure 6:**
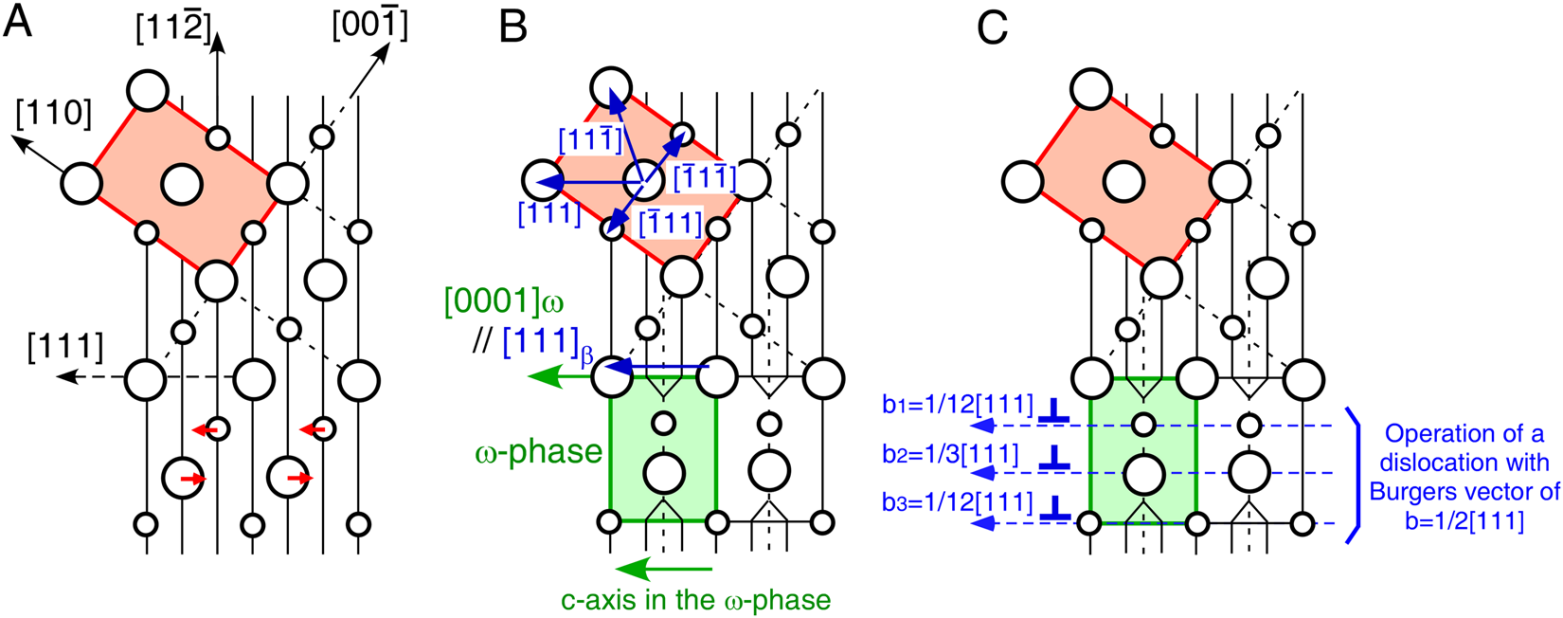
Possible model on the variations in atomic arrangement in bcc β-Ti during the dynamic precipitation of ω-phase. The observed direction is parallel to the [$\bar{1}$10] direction, and the large and small circles indicate the atoms in the plane of the paper and in the lower plane, respectively. (**A**) ω-phase formation in the β-matrix phase is crystallographically associated with shifts of atoms along opposite <111> directions, as indicated with red arrows. (**B**) During formation of the ω-phase, the atomic arrangement is conserved in only one of the four <111> directions, i.e., only the [111] direction. (**C**) According to the model proposed by Lasalmonie and Chaix^36^, if the dislocation with the Burgers vector of *b* = 1/2[111] is dissociated into the three *b*/6, 2*b*/3, and *b*/6 partial dislocations involving a three-layer fault in a dislocation core level, the dissociation can produce the same atomic arrangement of the β/ω-phase interface.

The origin of this unique phenomenon is considered to be related to the geometric relationship between the atomic arrangement of the bcc β-Ti matrix phase and the ω-phase. Figure 6 shows the atomic arrangement in bcc β-Ti observed along the [$\bar{1}$10] direction. The formation of the ω-phase is crystallographically associated with shifts of the atoms along opposite <111> directions, the coupling of which leads to the formation of the ω-phase through the collapse of two specific (111) layers, as shown in Fig. 6A and B. As another possible ω-phase formation process, Lasalmonie and Chaix proposed that if a dislocation with a Burgers vector of *b* = 1/2[111] is dissociated into three *b*/6, 2*b*/3, and *b*/6 partial dislocations involving a three-layer fault in a dislocation core level, the dissociation could produce the same atomic arrangement of the β/ω-phase interface as shown in Fig. 6C, which would induce formation of the ω-phase^36^. However, such dislocation dissociation has not been confirmed experimentally. Note that the ω-phase induced by this dislocation dissociation has a *c*-axis parallel to the Burgers vector of the dislocation, which is in good agreement with the present observations. Thus, it is possible that the stress-field formed under the to-and-fro motion of the dislocations assists in the above-described dislocation dissociation, although the details of this process must be clarified by further studies. During the formation of the ω-phase via the above mechanism, only the atomic arrangement along the [111]_β_ direction is conserved, unlike those along the other three of the four <111>_β_ directions, as shown in Fig. 6B. This is connected to the reason why the dislocation movement parallel to the *c*-axis of the ω-phase does not induce hardening, but rather causes softening.

In summary, we found a curious cyclic softening phenomenon that occurs in Ti-35Nb-10Ta-5Zr biomedical bcc β-Ti alloys, in which to-and-fro motion of a dislocation induces the precipitation of a ω-phase during cyclic deformation. This is the first report of the occurrence of a “dynamic precipitation softening” phenomenon in alloys. In addition, the present results provide new insight into the features of the ω-phase in β-Ti alloys. The ω-phase is generally believed to be a strong strengthening phase that simultaneously causes embrittlement of the alloy, but this is not always true. In fact, the ω-phase can induce strong softening of β-Ti alloys along a specific direction if its crystal orientation is controlled. The unique anisotropic mechanical properties obtained by the cyclic-deformation-induced oriented ω-phase formation may be useful for the development of a “single-crystalline β-Ti implant material” with advanced mechanical performance beyond the usual trade-off relationship between strength, Young’s modulus, and ductility via the appropriate crystal orientation control. We first found and clarified the mechanism of “dynamic precipitation softening” in alloys.

## Materials and Methods

Mother ingots with a composition of Ti-35Nb-10Ta-5Zr (wt. %) were prepared by melting high-purity Ti, Nb, Ta, and Zr in a plasma arc furnace. Single crystals were grown using the floating zone method under Ar flow at a growth rate of 2.5 mm/h. After the crystal growth, the crystals were slowly cooled to room temperature for 40 h. The obtained single crystals were enclosed in quartz capsules under Ar atmospheres and then subjected to solution treatment at 1063 K for 3.6 ks, followed by quenching into iced water. The chemical composition of the obtained single crystals was analyzed using an inductively coupled plasma atomic emission spectrometer (ICP-AES) and an oxygen analyzer (Kobelco Research Institute, Inc.), which confirmed that there was almost no change in the alloy composition relative to that of the mother ingot (see Supplementary Table S2). For the cyclic deformation test, specimens in the form of plates with gauge dimensions of 2 × 3 mm^2^ × 7 mm were cut by spark machining. The loading axis of the specimens was usually set to [$\bar{1}$49], for which the Schmid factor of the (10$\bar{1}$)[111] slip was 0.500. Tests at the [012] loading axis were also conducted under the same conditions in order to examine the orientation dependence of the cyclic deformation behavior. For this orientation, the Schmid factors of the (10$\bar{1}$)[111] and (101)[11$\bar{1}$] slips had the same high value of 0.490 (see Supplementary Table S1 and Supplementary Fig. S7 for details). The specimens were mechanically and then electrolytically polished in a solution of 6 vol.% perchloric acid/35 vol.% butanol/59 vol.% methanol at −25 °C to remove the surface damage. Cyclic deformation tests were performed in a symmetrical tension/compression mode at a fixed total strain amplitude (Δε/2) of 1.1% in air at room temperature (RT) using a Shimadzu EHF-EDS-10L servo hydraulic machine. The strain was measured by using a clip-on extensometer at 5 mm intervals on the gauge of the specimens. All of the tests were initiated in a tension state, and loading was applied in a sine wave. The frequency of the cycle was set to give a “mean” strain rate of 4.0 × 10^–4^ s^−1^.

From the cyclically deformed specimens, small rectangular specimens with dimensions of ~1.0 × 1.5 mm^2^ × 1.8 mm and with the loading axis parallel to [$\bar{1}$49] or [1$\bar{9}$4], were cut out, and then compression tests were conducted at RT to clarify the variations in the orientation dependence of the yield stress due to the development of the ω-phase. Small specimens were also cut out from the specimens cyclically deformed for both 50 cycles and 600–725 cycles. The former contained almost exclusively dislocations, but ω-phases also developed in the latter. The yield stress of the small specimens prepared from the undeformed crystal was also examined to estimate the base value.

Slip traces introduced on the deformed specimens were observed to confirm the operative deformation mode using an optical microscope equipped with Normarski interference contrast. In addition, thin foils were prepared by Ar^+^ ion bombardment from the deformed specimen, and the deformation substructure was observed in a TEM (JEOL JEM-3010) operated at 300 kV.

## Electronic supplementary material

Supplementary information
