# Supplementary material for: Unusual dynamic precipitation softening induced by dislocation glide in biomedical beta-titanium alloys
Source: Sci Rep. 2017 Aug 14;7:8056. doi: 10.1038/s41598-017-08211-7 (PMC5556124; doi:10.1038/s41598-017-08211-7)
Supplement: Supplementary file 1 — Supplementary information [file 41598_2017_8211_MOESM1_ESM.pdf]

SUPPLEMENTARY INFORMATION
for

# Unusual precipitation softening induced by dislocation glide in biomedical beta-titanium alloys

Koji Hagihara[1], Takayoshi Nakano[2]* and Mitsuharu Todai[2]

[1] Department of Adaptive Machine Systems, Graduate School of Engineering, Osaka University, 2-1 Yamadaoka, Suita, Osaka 565-0871, Japan
[2] Division of Materials and Manufacturing Science, Graduate School of Engineering, Osaka University, 2-1 Yamadaoka, Suita, Osaka 565-0871, Japan

* correspondence to: nakano@mat.eng.osaka-u.ac.jp

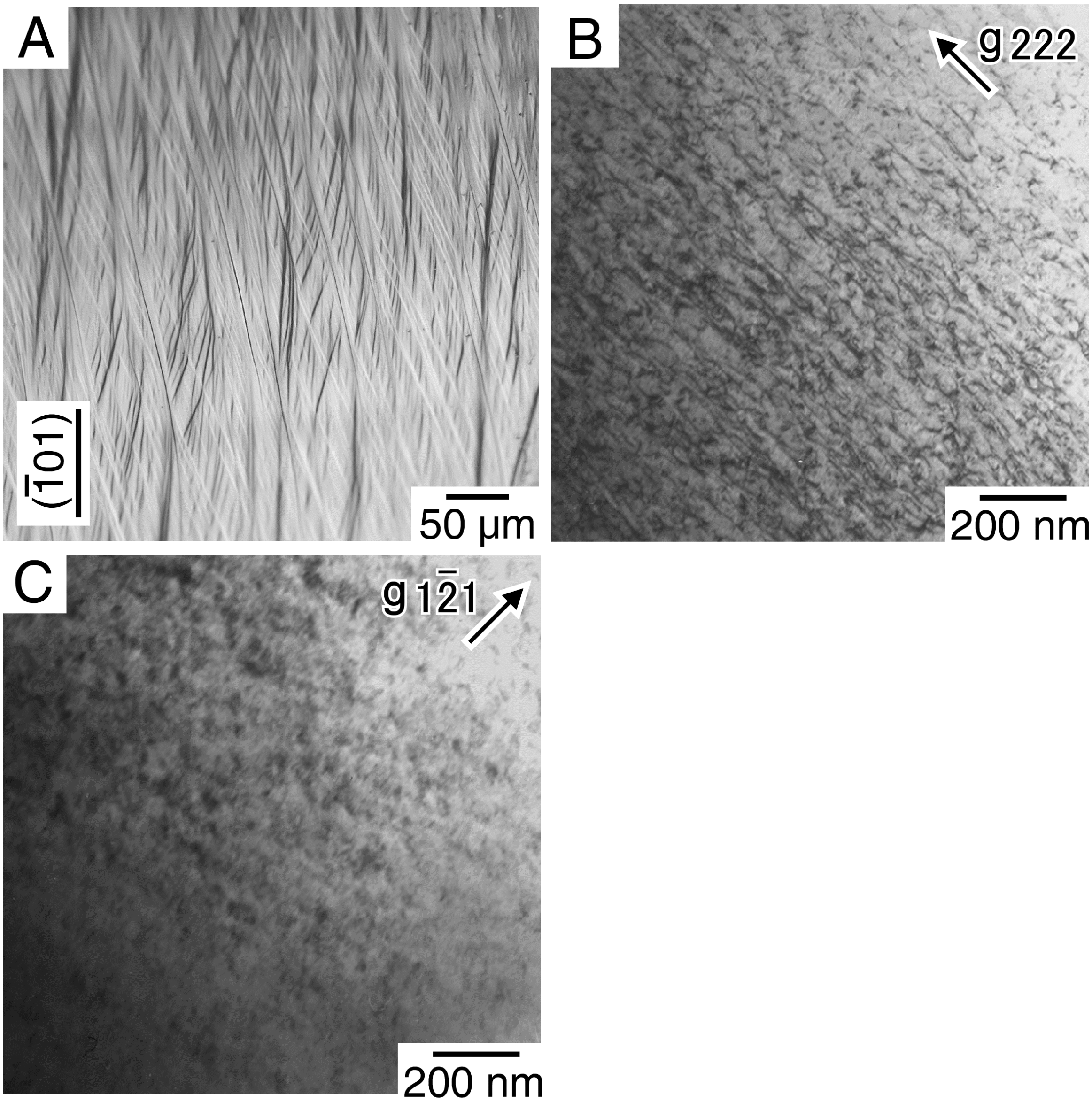


**Supplementary Figure S1 | Dislocations introduced in the specimen cyclically deformed at [ $\bar{1}$49] loading orientation. A,** Slip traces observed on the (11 5 $\bar{1}$) specimen surface cyclically deformed at $\Delta\varepsilon/2$ = 1.1% to 50 cycles. **(B, C)** TEM bright field images of the dislocations observed in the specimen cyclically deformed at $\Delta\varepsilon/2$ = 1.1% to 920 cycles. Beam // [10$\bar{1}$]. Dislocations were visible with **B,** g = 222 but lost their contrast with **C,** g = 1 $\bar{2}$ 1. The observation results demonstrate that dislocations with the Burgers vector parallel to [111] were operative as screw dislocations.

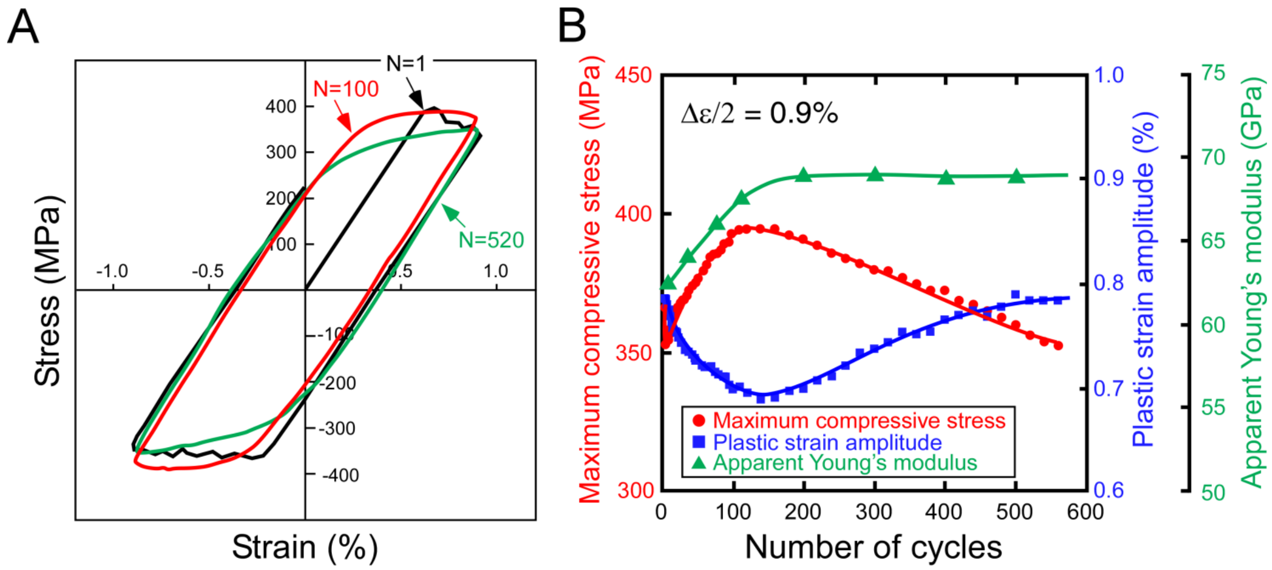


**Supplementary Figure S2 | Cyclic deformation behavior of a Ti-50Nb (wt. %) binary single crystal in which [111] dislocations are operative. A,** Typical hysteresis loops for a controlled total strain amplitude Δε/2 of 0.9% applied at the [$\bar{1}$49] loading orientation. **B,** Variations in maximum stress, plastic strain amplitude, and apparent Young's modulus with the number of cycles. Similar cyclic softening behavior as that observed in the Ti-35Nb-10Ta-5Zr alloy single crystal was observed.

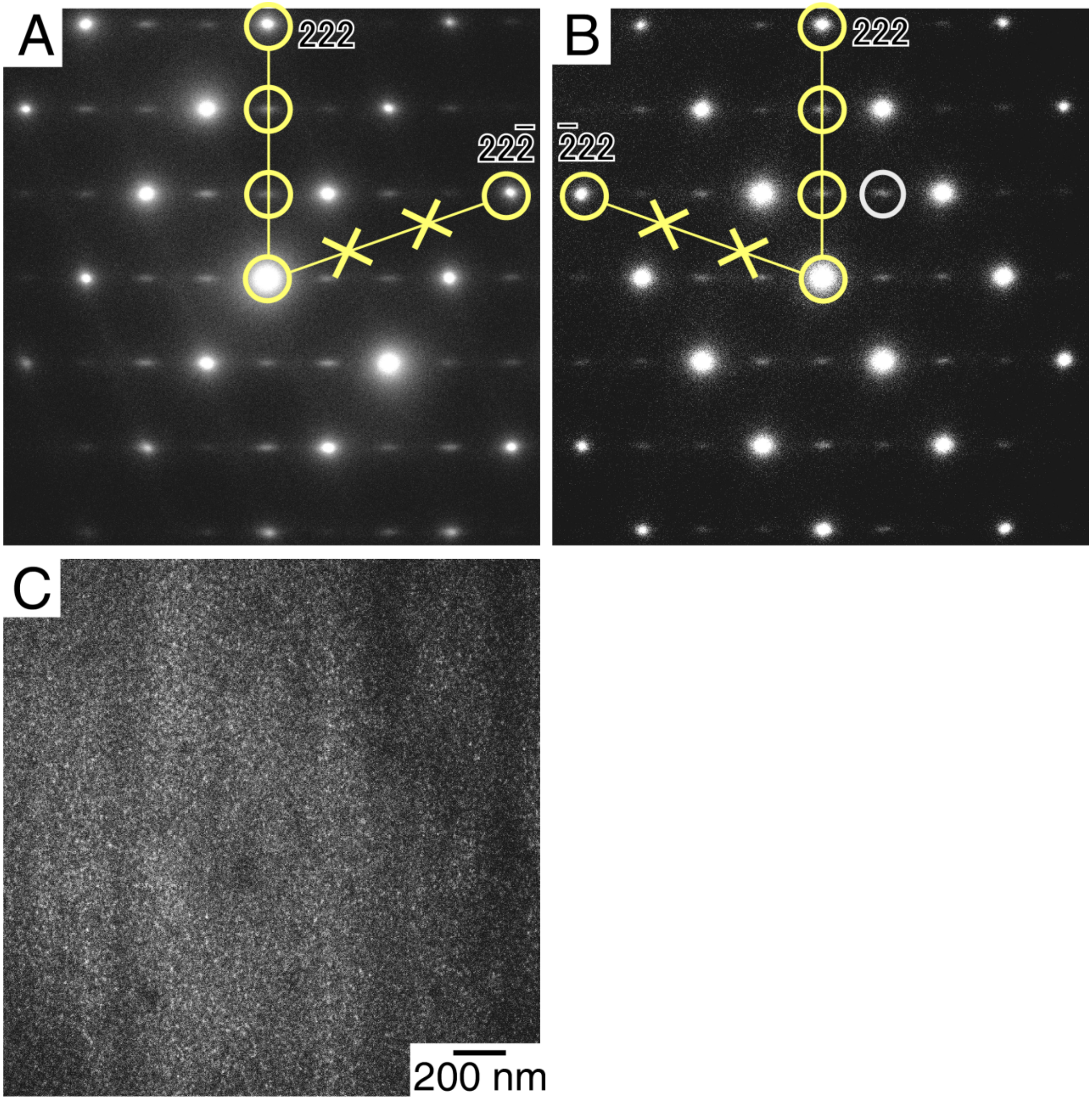


**Supplementary Figure S3 | ω-phase formation induced by the cyclic deformation in Ti-50Nb binary single crystal. (A, B),** SAED patterns of the specimen cyclically deformed at $\Delta\varepsilon/2 =$ 0.9% to 560 cycles, observed along the **A,** [$\bar{1}$10] and **B,** [01$\bar{1}$] directions, respectively. **C,** Dark-field image showing the abundant precipitation of the ω-phase, observed along the [01$\bar{1}$] direction. The extra spot used for the observation is denoted by the white circle in the SAED pattern shown in Fig. S3B.

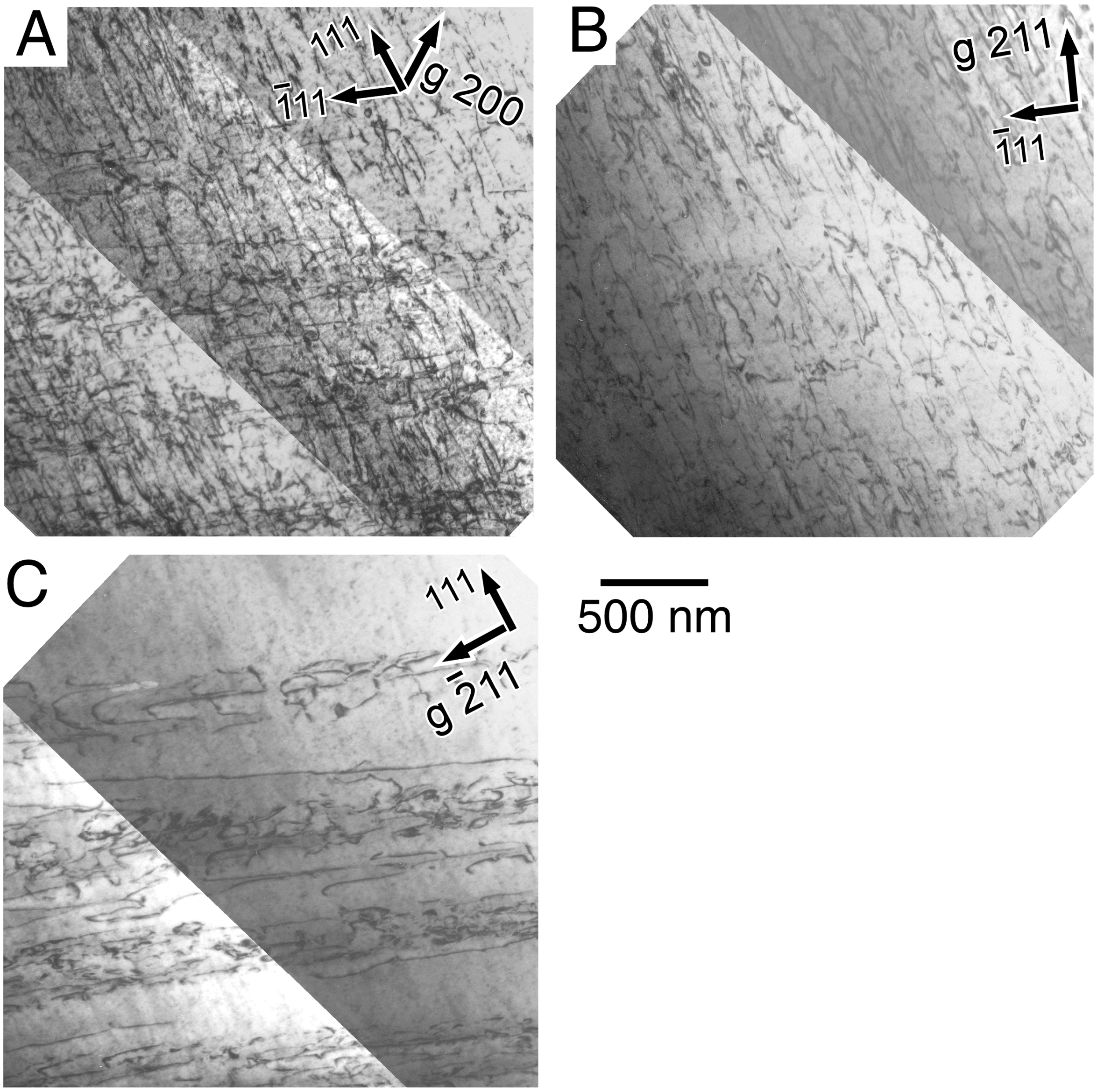


**Supplementary Figure S4 | Dislocations introduced in the specimen cyclically deformed at the [012] loading orientation in Ti-35Nb-10Ta-5Zr alloy single crystal.** The Burgers vectors of the dislocations were determined by g·b contrast analysis in the TEM, using the specimen deformed at $\Delta\varepsilon/2 =$ 1.1% to 830 cycles. Beam//[01$\bar{1}$]. Dislocation observation was conducted with **A**: g = 200, **B**: g = 211, and **C**: g = $\bar{2}$11. The observation results demonstrate that two kinds of dislocations with the Burgers vector parallel to [111] and [$\bar{1}$11] were operative as screw dislocations.

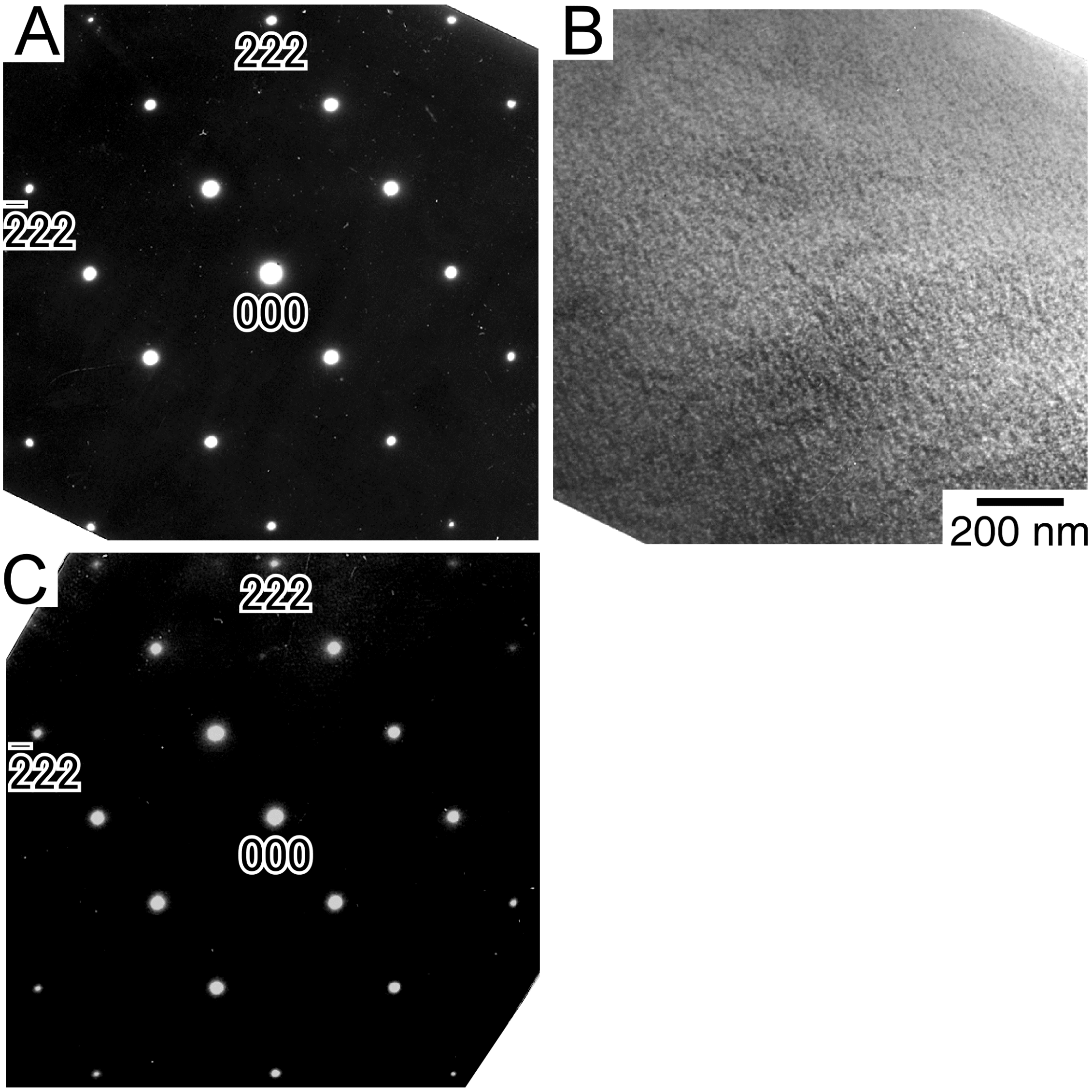


**Supplementary Figure S5 | Variations in SAED patterns with the deformation conditions at $[\bar{1}49]$ orientation in Ti-35Nb-10Ta-5Zr alloy single crystal. A,** SAED pattern observed in the specimen cyclically deformed at $\Delta\varepsilon/2 = 0.7\%$ to N = 9000, in which the cyclic deformation was carried almost exclusively by elastic deformation. No precipitation of the $\omega$-phase was observed in the specimen. **B,** Corresponding bright field TEM image of the microstructure. Few dislocations were observed in the specimen. **C,** SAED pattern observed in the specimen cyclically deformed at $\Delta\varepsilon/2 = 1.1\%$ to N = 50, i.e. before softening. The observed directions are all parallel to the $[01\bar{1}]$ direction. Precipitation of the $\omega$-phase was hardly observed in the specimen before cyclic softening occurred.

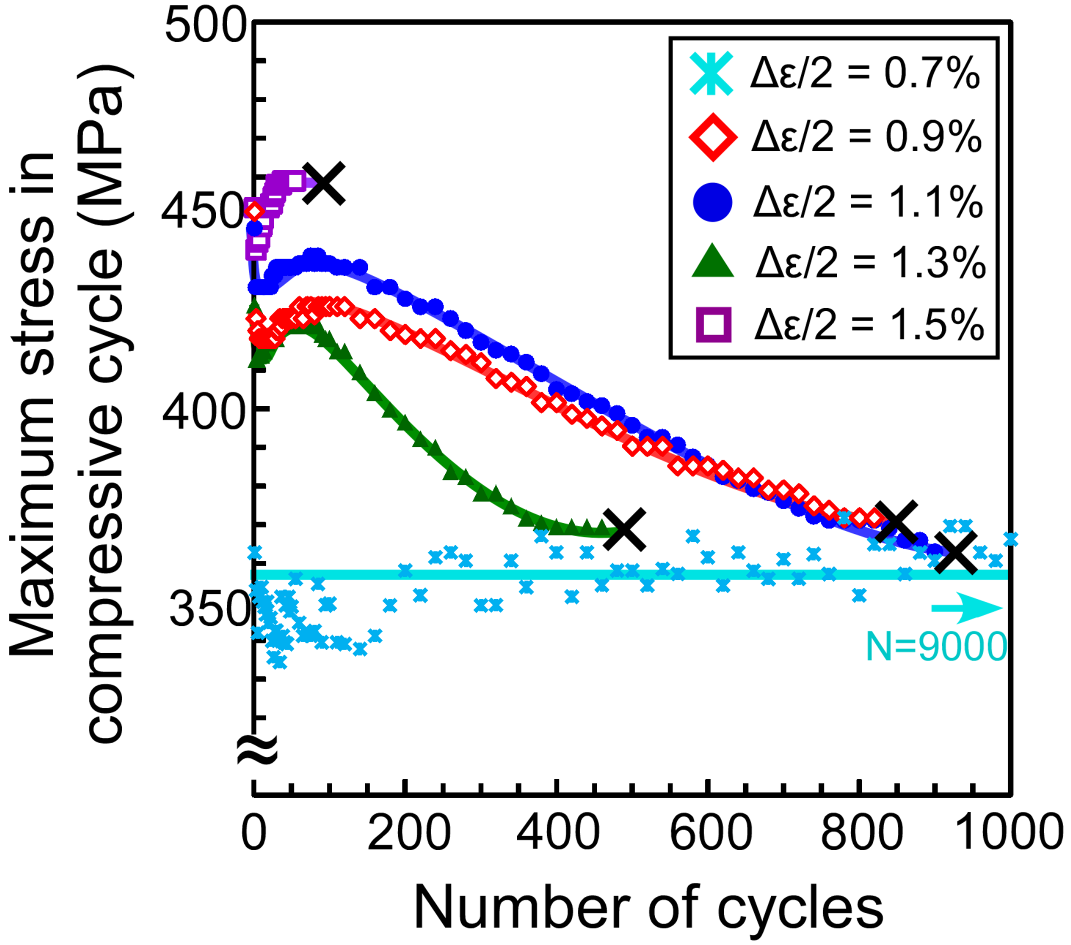


**Supplementary Figure S6 | Variations in maximum stress in compressive cycle with the applied total strain amplitude, in the cyclic deformation tests of Ti-35Nb-10Ta-5Zr alloy single crystals at [$\bar{1}$49] orientation.** The cyclic softening appeared in all of the specimens in which plastic deformation occurred, although facture occurred before softening in the specimen deformed at $\Delta\varepsilon/2 =$ 1.5%. On the other hand, no cyclic softening appeared in the specimen deformed at $\Delta\varepsilon/2 =$ 0.7%, in which cyclic deformation was carried almost exclusively by elastic deformation, as indicated in Supplementary Fig. S5B.

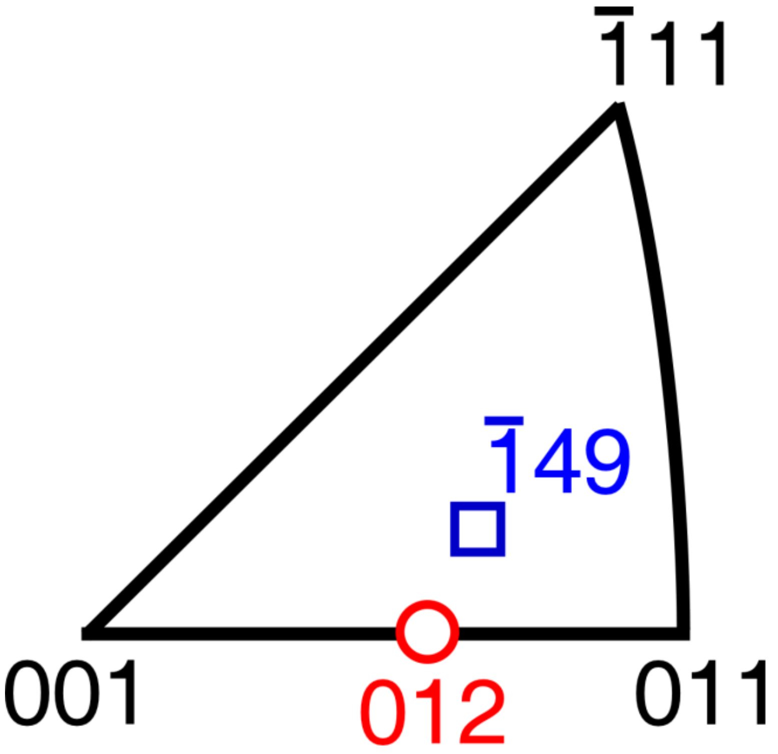


**Supplementary Figure S7 | Stereographic projection showing the [$\bar{1}$49] and [012] loading orientations investigated in the cyclic deformation tests in this study.**

**Supplementary Table S1 | Schmid factors for {101}<111> slip at the [$\bar{1}$49] and [012] loading orientations.**

| Slip system | Loading orientation | |
|---|---|---|
| | [$\bar{1}$49] | [012] |
| ($\bar{1}$0 1) [1 1 1] | 0.500 | 0.490 |
| (1 0 1) [$\bar{1}$ 1 1] | 0.467 | 0.490 |
| ($\bar{1}$ 0 1) [1 $\bar{1}$ 1] | 0.167 | 0.163 |
| (1 0 1) [1 1 $\bar{1}$] | 0.200 | 0.163 |
| ($\bar{1}$ 1 0) [1 1 1] | 0.250 | 0.245 |
| (1 1 0) [$\bar{1}$ 1 1] | 0.175 | 0.245 |
| (1 1 0) [1 $\bar{1}$ 1] | 0.050 | 0.082 |
| ($\bar{1}$ 1 0) [1 1 $\bar{1}$] | 0.125 | 0.082 |
| (0 $\bar{1}$ 1) [1 1 1] | 0.250 | 0.245 |
| (0 $\bar{1}$ 1) [$\bar{1}$ 1 1] | 0.292 | 0.245 |
| (0 1 1) [1 $\bar{1}$ 1] | 0.217 | 0.245 |
| (0 1 1) [1 1 $\bar{1}$] | 0.325 | 0.245 |

**Supplementary Table S2 | Chemical compositions of the grown Ti-Nb-Ta-Zr alloy single crystal examined in this study.**

| | Ti | Nb | Ta | Zr | O | N |
|---|---|---|---|---|---|---|
| Composition (wt.%) | bal. | 35.5 | 10.5 | 4.9 | 0.07 | 0.04 |
